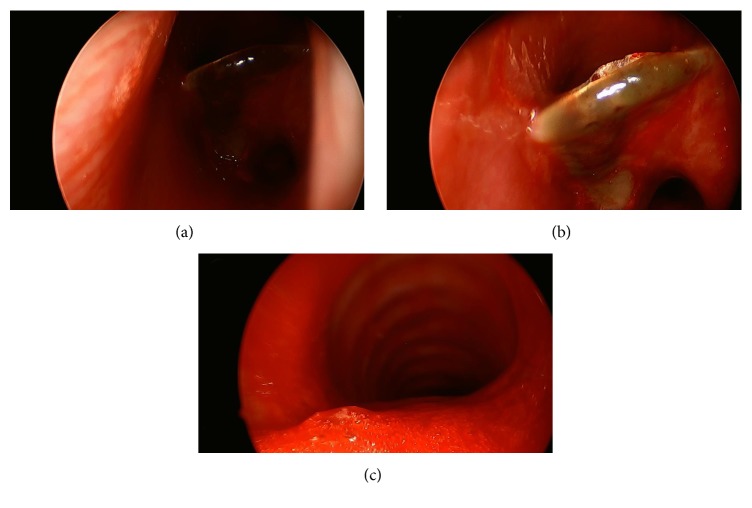# Aspiration of Aluminum Beverage Can Tab: Case Report and Literature Review

**DOI:** 10.1155/2017/1010975

**Published:** 2017-05-28

**Authors:** Alhasan N. Elghouche, Brian C. Lobo, Jonathan Y. Ting

**Affiliations:** Department of Otolaryngology-Head & Neck Surgery, Indiana University School of Medicine, Indianapolis, IN, USA

## Abstract

We describe the case of a 16-year-old male who aspirated a beverage can tab resulting in significant functional impairment. Since the introduction of beverage can opening tabs (“pop-tops” or “pull-tabs”) nearly 50 years ago, five cases of their aspiration have been reported in the literature and this is the first case to report tracheal lodgment. We describe the clinical course for this patient including the inadequacy of radiographic evaluation and a significant delay in diagnosis. We highlight unique features of small aluminum foreign bodies that require consideration and mention a potential change in epidemiology associated with evolving product design. Our primary objective is increased awareness among otolaryngologists that radiography is unreliable for diagnosis or localization of small aluminum foreign bodies. The patient history must therefore be incorporated with other imaging modalities and/or endoscopic evaluation. Also, given the marked prevalence of aluminum beverage cans, we suspect that the inadvertent aspiration of can tabs is more common than indicated by the paucity of published reports.

## 1. Introduction

**Figure 1:**
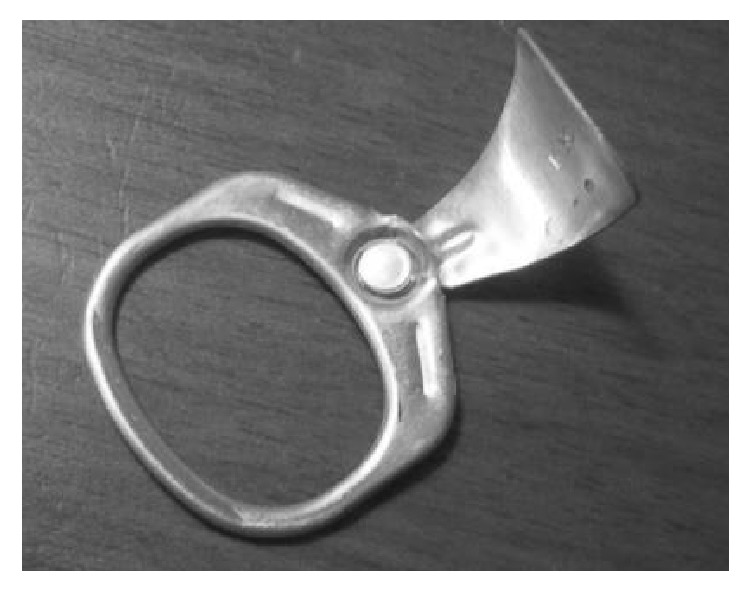


**Figure 2:**
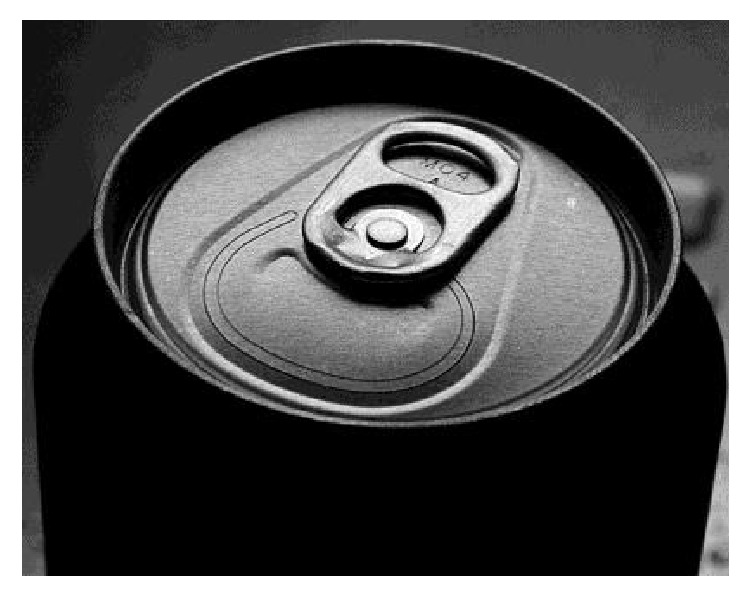


The first beverage cans were opened via puncture with a “church key” can opener. In the 1960s, the pull-tab mechanism was introduced. A pull-tab consists of a metal ring which, along with a wedge-shaped portion of the can top, is completely separated from the can when pulled to create an opening (Figure 1). Not long after their introduction, cases of accidental pull-tab ingestion and aspiration were described in the medical literature [1]. Their emergence as foreign bodies in conjunction with the rampant littering of these detachable tabs facilitated development of the presently employed “stay-tab,” meant to remain attached following can opening (Figure 2). While the stay-tab appears to have reduced litter, there are still reported injuries related to these small foreign bodies, more commonly in the context of ingestion as opposed to aspiration [2–4].

An important consideration in the aspiration of beverage can tabs is their aluminum composition. Despite being a metal, aluminum is relatively radiolucent and may evade radiographic detection [5]. In fact, this relative radiolucency influenced the United States Treasury decision to replace copper pennies with zinc instead of aluminum given the tendency of coins to become foreign bodies in the pediatric population [6].

## 2. Case Presentation

A previously healthy 16-year-old male presented to his primary care provider with the chief complaint of dyspnea at rest and on exertion. Associated symptoms included halitosis and foreign body sensation within the neck. He denied any cardiac symptoms, dysphagia, and odynophagia. His symptoms progressed such that the patient was unable to participate in gym class at school and he ultimately developed two-pillow orthopnea, sleep disturbance, and significant anxiety.

**Figure 3:**
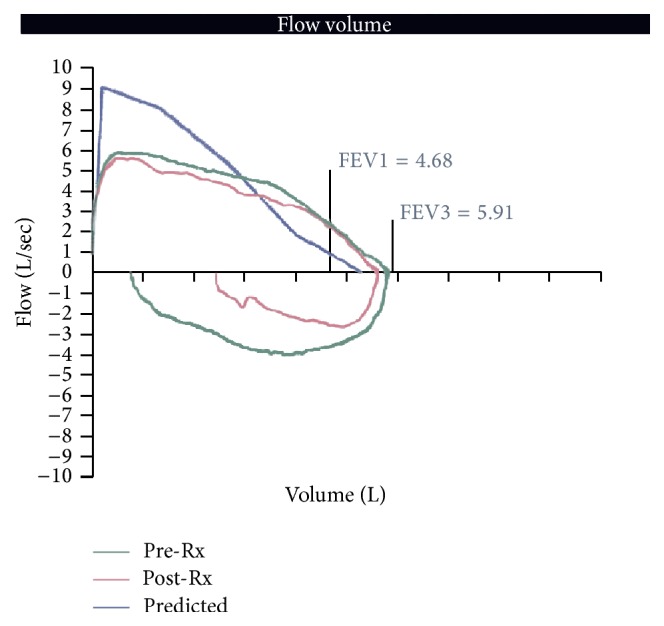


Physical examination was remarkable for increased pressure sensation upon palpation of the anterior neck inferior to the cricoid cartilage. The patient reported no relief with inhaled bronchodilators. Posteroanterior and lateral radiography was unremarkable. A noncontributory cardiac evaluation (including echocardiography) was followed by pulmonary function testing suggestive of fixed upper airway obstruction (Figure 3). During this time, the patient recalled an episode coincident with symptom onset in which he chewed and subsequently aspirated the opening tab from an aluminum soda can.

Bronchoscopic evaluation by the pulmonology team was attempted and a foreign body was noted immediately distal to the subglottis. At this point, the Otolaryngology-Head and Neck Surgery Service was consulted for further management. That evening, the patient was taken to the operating room for rigid laryngoscopy and bronchoscopy. After induction of generalized mask anesthesia and unremarkable assessment of the upper airway, a rigid bronchoscope was passed through the glottis to reveal a metallic foreign body in the proximal trachea with overlying mucoid debris (Figures 4(a) and 4(b)). Utilizing a Benjamin-Lindholm laryngoscope, optical forceps were passed through the glottis into the trachea to gently rotate and remove the lodged soda can tab. Superficial mucosal lacerations were seen at the site, with no evidence of granulation tissue or exposed cartilage (Figure 4(c)). The patient emerged from general anesthesia and was observed overnight. He reported symptom resolution and, following an uncomplicated postoperative course, was discharged the following day.

## 3. Discussion

Since 1975, five instances of aspiration of a beverage can tab have been described in the literature. We describe the first reported instance of lodging of an aspirated beverage can tab in the trachea. Timely integration of the patient history with appropriate diagnostic studies and interventions is necessary to avoid life-threatening sequelae [7].

Radiographic evaluation of the patient with this type of aspiration can be misleading: in our case leading to more than four months of misdiagnosis and an extensive cardiac and pulmonary diagnostic evaluation. One patient experienced ten years of cough and recurrent pulmonary infiltrates due to aspiration of a pull-tab into his left main stem bronchus which evaded detection by chest radiography [8]. A retrospective study determined that radiographic detection of can tabs was demonstrated in 20 percent of cases, only when an ingested tab was localized to the stomach [3]. When utilizing imaging studies in the workup of possible aspiration of a can tab, computed tomography in lieu of plain film has proven to be more beneficial [2]. In the above case, misrecognition of the radiographic properties of aluminum may have contributed to delayed diagnosis and an extensive, unnecessary diagnostic workup including echocardiography and pulmonary function testing. Further impeding radiographic diagnosis was the anatomic location of the foreign body given that 80% of laryngotracheal foreign bodies do not appear on X-ray [9].

Inadvertent ingestion of beverage can tabs is more frequently described than aspiration. This is perhaps due to a tendency to place a detached tab into the contents of the can while drinking. Interestingly, the design change from pull- to stay-tabs has potentially affected the patient population at risk. The eldest of seven patients described in a 1976 case series of tab ingestions and aspirations (prior to the advent of the stay-tab) was two years old [10]. In contrast, the majority of patients in a 2010 study of inadvertent ingestion were teenagers [3]. A potential explanation is reduced access by infants and young children to tabs that remain attached to beverage cans.

Focusing specifically on aspiration. during the pull-tab period, two out the three reported aspirations occurred in infants [10]. Despite the small sample size, following the introduction of the stay-tab design, there have been no reported cases of aspiration in children. From a product design standpoint, this may indicate the successful mitigation of a pediatric safety hazard and resultant change in the population comprising aspiration of beverage can tabs. Of the described instances of beverage can tab aspiration, one was found at the glottis, one at the carina, and the remainder within the bronchi [1, 4, 8, 10].

The evolving design of beverage can tabs illustrates product changes that attempt to enhance patient safety. The capacity for inadvertent ingestion or aspiration does remain; however, depending on the actual extent of ingestion or aspiration, increased consumer awareness or another evolution in product design may be warranted. Potential solutions include introduction of a design that further limits detachment from the can or use of a more radiodense material for the opening tab component.

## 4. Conclusion

Beverage can tabs continue to act as foreign bodies though perhaps in an older patient population subsequent to their design change. Though ingestion appears to be more common, aspiration continues to occur. Despite being metal, X-ray investigation provides poor negative predictive value in evaluation for aluminum beverage can tab, especially in cases of suspected aspiration. Alternate imaging modalities or endoscopy should be pursued to establish a timely diagnosis and avoid secondary injury.

## Figures and Tables

**Figure 4:**